# Towards understanding the gliotoxin detoxification mechanism: *in vivo* thiomethylation protects yeast from gliotoxin cytotoxicity

**DOI:** 10.15698/mic2016.03.485

**Published:** 2016-02-19

**Authors:** Elizabeth B. Smith, Stephen K. Dolan, David A. Fitzpatrick, Sean Doyle, Gary W. Jones

**Affiliations:** 1 Department of Biology, Maynooth University, Maynooth, County Kildare, Ireland.

**Keywords:** gliotoxin, Aspergillus fumigatus, Saccharomyces cerevisiae, oxidoreductase GliT, thio-methyltransferase GtmA

## Abstract

Gliotoxin (GT) is a mycotoxin produced by some species of ascomycete fungi including the opportunistic human pathogen *Aspergillus fumigatus*. In order to produce GT the host organism needs to have evolved a self-protection mechanism. GT contains a redox-cycling disulfide bridge that is important in mediating toxicity. Recently is has been demonstrated that *A. fumigatus* possesses a novel thiomethyltransferase protein called GtmA that has the ability to thiomethylate GT *in vivo*, which aids the organism in regulating GT biosynthesis. It has been suggested that thiomethylation of GT and similar sulfur-containing toxins may play a role in providing self-protection in host organisms. In this work we have engineered *Saccharomyces cerevisiae*, a GT-naïve organism, to express *A. fumigatus* GtmA. We demonstrate that GtmA can readily thiomethylate GT in yeast, which results in protection of the organism from exogenous GT. Our work has implications for understanding the evolution of GT self-protection mechanisms in organisms that are GT producers and non-producers.

## INTRODUCTION

Gliotoxin (GT) is a fungal natural product with known antibiotic and antifungal properties and is biosynthesized by the opportunistic human pathogen, *Aspergillus fumigatus*, as well as by related ascomycetes 1,2,3. Gliotoxin biosynthesis is encoded by the *gli* gene cluster, which contains 13 genes, and the function of most of these genes has now been elucidated 4. Effectively, GT is the prototypic epipolythiodioxopiperazine (ETP) and is structurally and functionally related to a range of disulfide bridge-containing non-ribosomal peptides including, amongst others, sporidesmin A and sirodesmin 2,5. GT is redox-active; thus, cycling between the oxidized and reduced dithiol form (GT-(SH)_2_) can generate reactive oxygen species, which have deleterious cellular effects. GT auto-induces its own biosynthesis by activating and maintaining *gli* cluster expression 6. In animal cells, GT can inactivate selected protein functionality by covalent interaction with protein thiols and also deplete cellular glutathione (GSH) 7,8. Indeed, the cytotoxicity of GT is such that an independently regulated gene within the *gli* cluster, *gliT* encodes GT oxidoreductase which recognizes GT-(SH)_2_ and catalyzes disulfide bridge closure and is essential in *A. fumigatus* for self-protection against exogenous GT 9,10,11.

GT is not biosynthesized by *Saccharomyces cerevisiae* (baker’s yeast). As a consequence, no endogenous protection system against GT exists in this species and *S. cerevisiae* growth can be inhibited by exposure to GT 10,12,13. Expression of the *A. fumigatus* *gliT*-mediated self-protection system in yeast has been shown to confer GT resistance, as has deletion of *GSH1*, responsible for GSH biosynthesis 10,12. Thus, by enabling disulfide bridge closure, preventing the GSH-mediated chemical reduction of exogenously-added GT to GT-(SH)_2 _or facilitating GT efflux, the resistance of gliotoxin-naïve species to this redox-active molecular species can be augmented.

**Figure 1:**
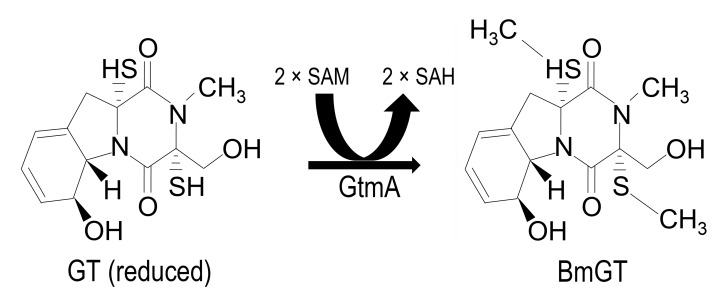
FIGURE 1: Conversion of reduced GT to BmGT is mediated by GtmA protein. Reduced GT undergoes *bis*-thiomethylation via the action of GtmA protein and requires two molecules of SAM for the reaction to reach completion 14. Consequently two molecules of SAH are produced that re-enter the methyl-methionine cycle 17.

Gliotoxin* bis*-thiomethyltransferase (GtmA), the first *bis* thiomethyltransferase so far identified in any species, has recently been described in *A. fumigatus* and it has been shown to convert GT-(SH)_2_ to bisdethiobis(methylthio)gliotoxin (BmGT) (Figure 1) 14. GtmA has also been contemporaneously characterized by others, where it is referred to as TmtA 15. Interestingly, although GtmA is encoded outside the *gli* cluster, its expression is induced by GT and it has been convincingly demonstrated to dissipate GT levels thereby attenuating *gli* cluster activity and concomitant GT biosynthesis 14. In effect, GtmA appears to be the ‘off-switch’ for GT biosynthesis in *A. fumigatus*. Although an unidentified thiomethyltransferase has been shown to confer resistance to a bacterial non-ribosomal peptide, holomycin, in* Streptomyces clavivulgaris* 16, *gtmA* deletion has effectively no impact on the sensitivity of *A. fumigatus* to GT 14,15.

Expression of *gtmA* in *S. cerevisiae* was therefore undertaken to further investigate the functionality of this enzyme and to explore the hypothesis that, in the absence of concomitant GT biosynthesis and a* gliT*-mediated self-protection system, GtmA could confer resistance to GT via BmGT formation.

## RESULTS AND DISCUSSION

### Expression of *gtmA* in yeast increases resistance to gliotoxin

**Figure 2:**
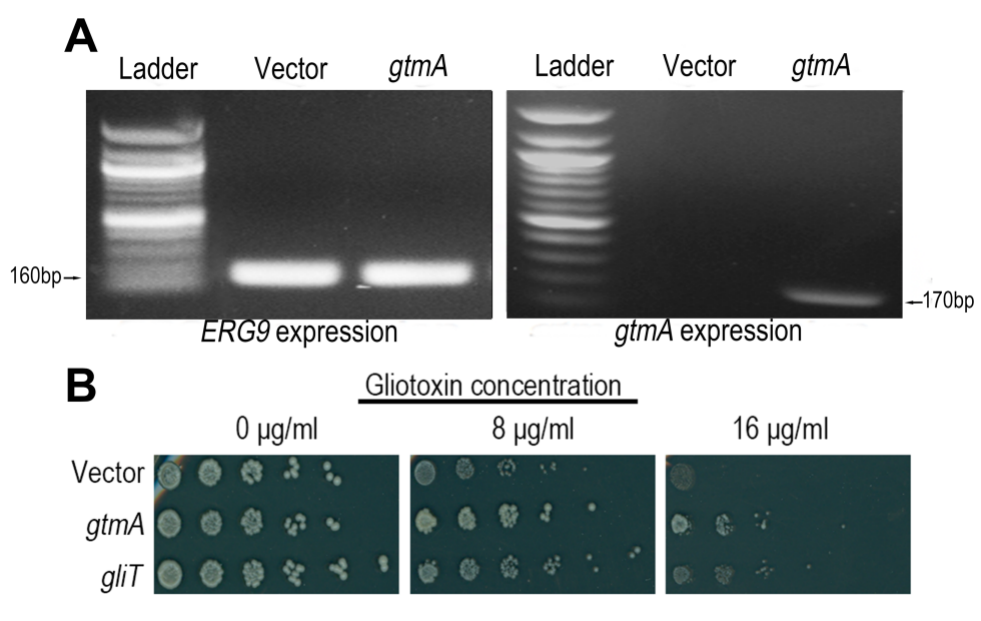
FIGURE 2: Expression of *gtmA* in yeast protects against GT cytotoxicity. **(A)** RT-PCR demonstrating that *gtmA* mRNA is stably expressed in yeast. Yeast *ERG9* mRNA production is shown as a positive control. **(B)** Expression of *A. fumigatus* genes *gtmA* or *gliT* in yeast provides protection against GT cytotoxicity.

While it has been clearly demonstrated that GliT provides the primary self-protection mechanism against GT in *A.* *fumigatus* 9,10 and that the production of BmGT via GtmA is primarily linked to GT biosynthesis and regulation 14, given that the production of BmGT will nullify GT ability to redox cycle, we hypothesized that under certain biological conditions GtmA may be able to provide direct protection against GT toxicity. In previous work from our group and others 10,12,13 yeast was utilized as a tool to explore the *in vivo* effects of exposure to GT. Following cloning of *gtmA* under control of the constitutive *SSA2* promoter we demonstrated that *gtmA* is well expressed in yeast (Figure 2A). Growth of yeast is severely impaired on medium containing gliotoxin (8 and 16 μg/mL, respectively), but expression of *gtmA* in yeast provides protection against this cytotoxicity (Figure 2B). The level of protection provided to yeast by *gtmA* is more prominent than that provided by *gliT* expression (Figure 2B). Additionally, the level of protection provided when both *gliT* and *gtmA* are expressed in yeast is comparable to *gtmA* expression alone, which reflects the apparent more efficient GT detoxification of GtmA compared to GliT when engineered in yeast. The expression of both *gtmA* and *gliT* does not allow yeast to efficiently grow on GT concentration above those used in this study [data not show]. Expression of the other methyltransferases encoded within the *gli*-cluster, *gliM* and *gliN* 15, did not provide any protection in yeast against GT toxicity (data not shown).

### Expression of *gtmA* in yeast causes BmGT formation from exogenously-added gliotoxin

**Figure 3:**
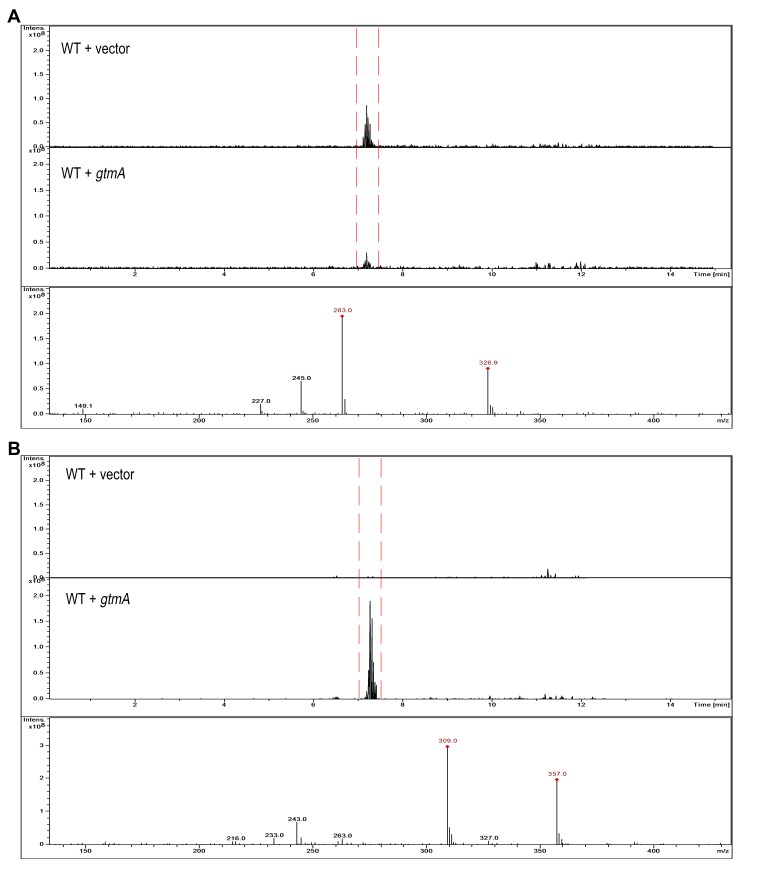
FIGURE 3: Detection and monitoring of GT and BmGT in yeast using mass spectrometry. **(A)** GT is readily detected in supernatants of yeast cells exposed to exogenous GT. **(B)** BmGT can only be detected in supernatants from yeast cells, which express *gtmA*, following exposure to exogenous GT.

**Figure 4:**
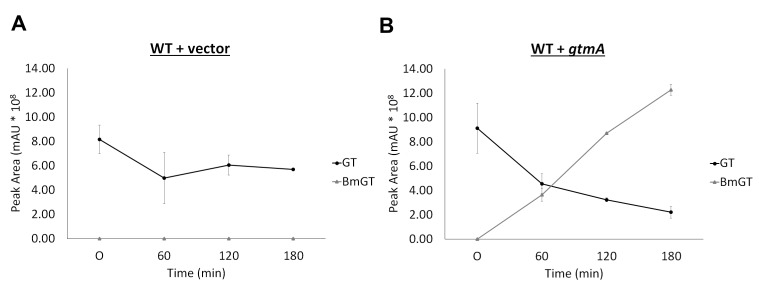
FIGURE 4: Conversion of GT to BmGT over time in yeast expressing GtmA. **(A)** BmGT is not detected in supernatants of yeast cells exposed to exogenous GT over 3 h exposure. **(B)** BmGT is readily detected in supernatants from yeast cells expressing *gtmA*. Increased levels of BmGT can be detected over a 3-hour time period and correlate with a diminution GT levels.

Yeast does not contain an ortholog or homolog of *A. fumigatus gtmA* 14. Consequently it is not surprising that we have never detected the production of BmGT in yeast following exposure to GT (Figure 3A and Figure 4A). The expression of *gtmA* in yeast provides this GT-naïve organism with the capability of converting GT to BmGT (Figure 3B). The production of BmGT in yeast expressing *gtmA* is as a result of utilizing exogenous GT as a substrate (Figure 4B).

### Biological relevance for gliotoxin conversion to BmGT 

What is the biological significance for GT to BmGT conversion? The results we present here clearly show that GT conversion to BmGT protects against cytotoxicity and therefore appear to support the suggestion that thiomethylation of GT is a protection mechanism, perhaps from exogenous and/or endogenously produced GT. However, to uncover the true biological relevance of GT to BmGT conversion we need to consider this reaction in the context of organisms that produce GT and those that do not. Recent evidence has clearly shown that deletion of *gtmA*, and hence the removal of the ability to convert GT to BmGT, in *A. fumigatus *does not produce a GT-sensitive phenotype 14,15. This is due to the maintenance of a fully functional, primary detoxification system, GliT, in a *gtmA* deletion strain. Furthermore, it has recently been demonstrated that GliT functions in conjunction with the GT-specific transporter GliA to maintain intracellular GT at a level that is not toxic to the host cell 18. The production of BmGT in *A. fumigatus* facilitates the secretion of this product in a GliA independent manner 18. The secretion of BmGT from fungal cells most likely occurs through a non-specific mechanism as the molecule is efficiently secreted from yeast (this study) and from GliA-deficient *A. fumigatus* 18. Thus the primary physiological relevance of converting GT to BmGT in GT-producing organisms appears to be as a negative regulator of GT biosynthesis, followed by secretion of the inactive thiomethylated derivative 18. However, the *in vivo* consequence of GtmA activity, and consequent BmGT production and secretion, will depend upon whether an organism is a GT producer or not.

Here we clearly show that engineering a GT to BmGT conversion mechanism into a GT naïve organism, such as yeast, can provide efficient protection against exogenous GT (Figures 2, 3 and 4). The yeast genome does not encode for any GtmA homologs and does not endogenously convert GT to BmGT (Figure 3). Previous phylogenetic analysis identified organisms within the fungal kingdom that possess GtmA homologs 14 and the implications from the results of this study suggest that a GT non-producing organism that possesses a GtmA homolog may well utilize such a protein as a defense mechanism against GT exposure. The absence of GT production in such organisms may suggest that from an evolutionary perspective such GtmA homologs may have evolved to carry out other GT-independent functions in the cell and, if they possess the ability to produce BmGT, this may not be the primary function of the protein. Conversely, GT non-producing fungi may have retained GtmA homologs as a means of occupying the same habitats as GT-producing fungi.

Moreover, the possibility does exist that GtmA homologs in GT naïve fungi constitute an ancient ETP generic-defense mechanism that has allowed the acquisition and development of the GT-producing gene cluster, or other ETP clusters. The evolution of the primary detoxification system involving GliT and GliA, in conjunction with the presence of GtmA, has then allowed for intricate systems-level interactions to develop in organisms such as *A. fumigatus*, as recently shown for GT biosynthesis and interplay with the methyl/methionine cycle 18 and for regulation of the *gli*-cluster itself 14. Any effect upon GT sensitivity of removing the GT to BmGT conversion system in *A. fumigatus*, or any GT-producing organism, may only become apparent when undertaken along with the concomitant abrogation of the primary GT-protection mechanism from the organism. Support for this hypothesis comes from the recent discovery that deletion of the *gtmA* homolog in GT-naïve *Aspergillus niger* results in sensitivity to exogenously added GT 19.

## Materials and Methods

### Yeast strains, plasmids and genetic methods

The *S. cerevisiae* strain used in this study was BY4741 (*MATa; his3*Δ* 1; leu2*Δ* 0; met15*Δ* 0; ura3*Δ* 0*) and was obtained from Euroscarf. All media used were as previously described by 20. Cultures were grown at 30°C with shaking at 200 rpm.

To observe the effects of *gtmA *expression in *S. cerevisiae, gtmA *was amplified from *A. fumigatus* cDNA (ATCC26933) using primers gtmA F and gtmA R (Table 1) and cloned into the yeast shuttle vector pC210 as previously described by 10. Briefly, primers were designed that incorporated *NdeI* and *SphI *restriction sites. Following PCR amplification of the *gtmA *gene, digestion of the *gtmA *PCR product and of pC210 was carried out followed by ligation using T4 DNA ligases (Promega) according to manufacturer’s instructions to create pC210-gtmA. Cloning of the *gtmA* fragment and construct were confirmed using Sanger sequencing.

**Table 1:** Oligonucleotide primers used in this study.

**Primer Name**	**Sequence 5’-3’**
gtmA F	AAAAAACATATGATGTCCAAGTCAGACTACATCCAG
gtmA R	AAAAAAGCATGCCTAGGGCTTGAGGCCGGTTG
ERG9 INTERAL F	GACTTATTTGGCCGGTATCCACG
ERG9 INTERNAL R	CTCGACACAGCCACGCAAAGTCC
gtmA RT-PCR F	TCCAGCGTACTCAACCACAC
gtmA RT-PCR R	CGTCTGGAAAGCTCTGGAA

Growth analysis was carried out by diluting an overnight culture of cells in fresh medium lacking leucine (-LEU) to an OD_600_ = 0.2 and incubated at 30°C shaking 200 rpm till an OD_600_ = 0.4 was reached. Cells were transferred to a microtiter plate and serially diluted. Cells were transferred to –LEU plates containing the desired concentration of GT (Sigma) using a replicator and incubated at 30°C for 48 h, with further monitoring at room temperature for 72 h.

### RT-PCR

Total RNA was extracted from 5 mL cultures of the yeast strains grown overnight at 30°C shaking 200 rpm. RNA was extracted using the QIAGEN RNeasy plant mini kit as per manufacturers’ guidelines. RNA was DNase treated using DNase I kit (Sigma-Aldrich), according to the manufacturer’s recommendations. RNA concentrations were measured using a Nano Drop 1000 Spectrophotometer (Mason Technology).

cDNA was synthesized using the qScript^TM^ cDNA Supermix (Quanta Biosciences) as per manufacturer’s instructions. Accu*Taq *LA polymerase (Sigma) was used to amplify a housekeeping gene *ERG9* as a control and the gene of interest *gtmA.* The primers used in RT-PCR reactions are listed in Table 1. PCR reactions were conducted in a PTC-200 Peltier Thermal Cycler (MJ Research) and the program consisted of 1 cycle of 95°C for 5 min, 35 cycles of 95°C for 30 sec, 58°C for 30 sec and 68°C for 30 sec. Followed by a further cycle of 68°C for 10 min.

### Mass spectrometry detection of gliotoxin and *bis*-methylgliotoxin

GT uptake and BmGT conversion assays were carried out by diluting overnight cell cultures in -LEU medium to an OD_600_ = 0.4 and incubation at 30°C shaking 200 rpm till an OD_600_ = 1.2 was reached, followed by GT (5 μg/ml) addition and incubation for a further 3 h. Samples were taken every 30 min during the 3 h period, and were then centrifuged at 5,000 x g to obtain the supernatant, which was then organically extracted using an equal volume of chloroform. Organic extracts were dried to completion under a vacuum and re-solubilized in methanol and analyzed by LC-MS (Agilent LC-MS system- Model 6340) as previously described 17.
